# Excellent survival of second-generation uncemented dual mobility cups compared with first-generation cups at a minimum of 10 years follow-up in primary total hip arthroplasty

**DOI:** 10.1051/sicotj/2024024

**Published:** 2024-08-27

**Authors:** Antoine Duhil, Gérald Delfosse, Elvire Servien, Cécile Batailler, Sébastien Lustig

**Affiliations:** 1 Department of Orthopedic Surgery, CHU Guadeloupe Pointe à Pitre France; 2 Department of Orthopedic Surgery and Sport Medicine, Croix-Rousse Hospital, FIFA Medical Center of Excellence Lyon France; 3 EA 7424, Interuniversity Laboratory of Human Movement Science, Université Lyon 1 Lyon France; 4 Université de Lyon, Université Claude Bernard Lyon 1, IFSTTAR, LBMC UMR_T9406 69622 Lyon France

**Keywords:** Dual mobility cup, THA, Aseptic loosening, Acetabular coating

## Abstract

*Introduction*: This study aimed to compare the revision rate and long-term survival between two generations of uncemented dual mobility cup (DMC) from the same manufacturer in primary total hip arthroplasty (THA) at a minimum follow-up of 10 years. *Methods*: This retrospective monocentric study included all THA performed with an uncemented DMC from the same company. The cohort included 150 patients with 22 first-generation DMC and 128 second-generation DMC. The coating of the second generation was a double-coating Plasma spray of Titanium and Hydroxyapatite (HAP), compared to the coating of alumina and HAP for the first generation. The mean follow-up was 14.2 ± 1.2 years. The mean age was 76.0 ± 10.1 years. The Harris hip score (HHS), complications, and revisions were collected at the last follow-up. Ten- and fifteen-year Kaplan-Meier survival was calculated. *Results*: At the last follow-up, the mean HHS was 83.2 ± 9.1. There were two acetabular loosenings with the old coating (9.1%) and one case with the new one (0.78%) (*p* = 0.056). There was one extra-prosthetic dislocation (0.67%) and one postoperative infection (0.67%). Survival without acetabular revision at 10 and 15 years was 90.9% for the 1st generation and 99.2% for the 2nd generation (*p* = 0.009). *Conclusion:* Survival without acetabular revision was significantly higher at 10 and 15 years of follow-up with the second generation of DMC with plasma-sprayed titanium and HAP coating compared to the first generation of DMC coat. The dislocation was uncommon, thanks to the dual mobility concept. This second generation of uncemented DMC can be safely used in primary THA.

## Introduction

Total hip arthroplasty (THA) is one of the most common surgeries in orthopedic departments. One of their most concerning complications was hip dislocation, with a rate reaching 7% in some cases. In the 70 s, Bousquet proposed a solution to address this issue with the dual mobility cup (DMC) [1, 2]. This innovation incorporated two principles: the first was based on McKee-Farrar’s concept, which enhances stability by utilizing a larger diameter head, and the second drew from Charnley’s principle of low friction [3]. The DMCs consist of two articulations: the small articulation between the femoral head and the polyethylene, and the large articulation between the polyethylene and the inner surface of the acetabular implant. This dual articulation allows for a greater range of motion and increased jump distance, reducing the risk of dislocation.

Despite these promising advantages, the use of DMC in primary intention remains limited and controversial. Indeed, several specific complications of DMC have been described, such as aseptic loosening and intra-prosthetic dislocation (IPD), particularly with first-generation DMCs [4, 5]. These complications were attributed to the design and coating of the first-generation implants (alumina and hydroxyapatite (HAP)). Consequently, manufacturers have developed new shapes and coatings for DMC, with a hydroxyapatite and titanium plasma spray coating. According to current literature, these enhancements in design and coating seemed to decrease the complication rate [6, 7]. However, no study has compared first- and second-generation DMCs from the same manufacturer. Moreover, the studies were often heterogeneous with difficulties to compare them together.

The objectives of this study were to compare the revision rate and long-term survival between two generations of uncemented DMCs from the same manufacturer in primary THA at a minimum follow-up of 10 years.

## Material and methods

### Patients

**Figure 1:**
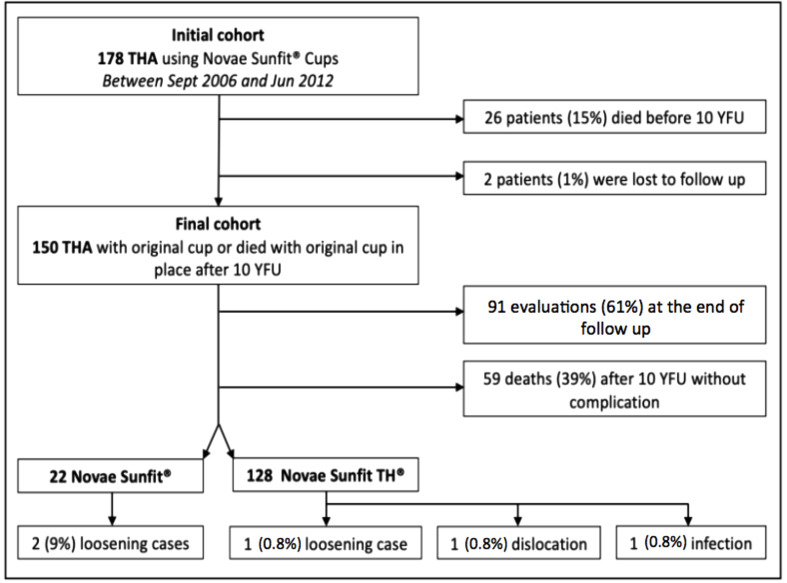
Flowchart.

**Figure 2:**
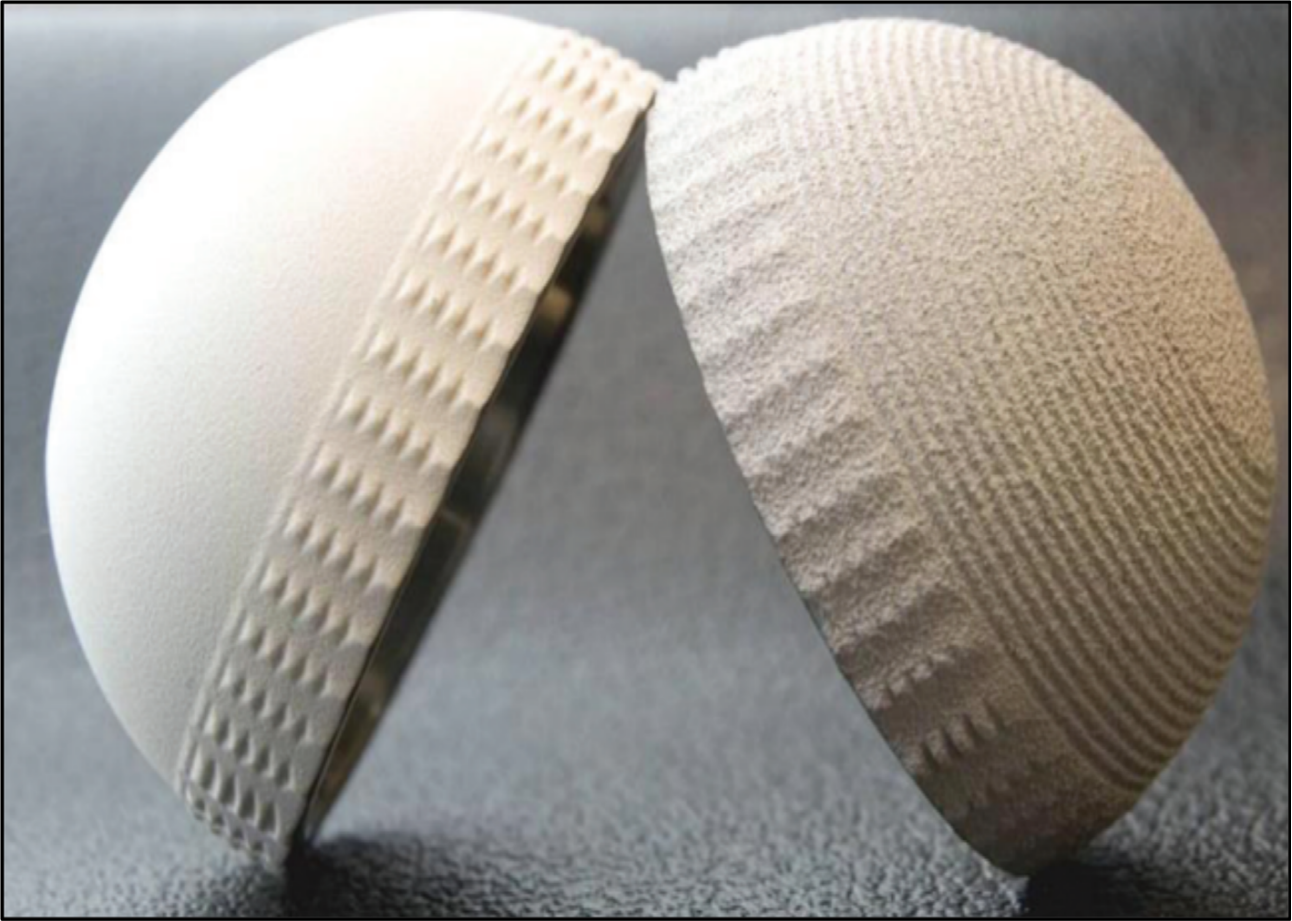
Sunfit^®^ cup 1st G (left) and Sunfit TH^®^ cup 2nd G (right).

This retrospective, monocentric, study included all primary THA using the uncemented DMC Novae Sunfit^®^ Serf between September 2006 and June 2012. The exclusion criteria were a follow-up inferior to 10 years. Of the 178 patients meeting these criteria, 26 patients died before 10 years with their DMC in place (15%), and 2 patients were lost to follow-up before 10 years (1%) (Figure 1). The cohort included 150 patients with 22 first-generation DMCs (Novae Sunfit^®^ Serf cups coated with HAP and alumina) and 128 second-generation DMCs (Novae Sunfit TH^®^ Serf cups coated with HAP and plasma spray titanium) (Figure 2).

The mean follow-up was 14.2 ±1.2 years (10.4–16.2), with no significant difference between the two groups. There were 43 men (28.7%) and 107 women (71.3%), with a mean age of 76.0 years ±10.1 (28–90).

Etiologies were primary osteoarthritis of the hip (*n* = 109, 73%), aseptic osteonecrosis (*n* = 15, 10%), neck fracture (*n* = 11, 7%), rapidly destructive osteoarthritis of the hip (*n* = 7, 5%), post-traumatic osteoarthritis (*n* = 5, 3%), and dysplasia (*n* = 3, 2%). The mean body mass index (BMI) was 27.3 ± 4.8 kg/m^2^ (18–43) (Table 1).

**Table 1:** Pre and intra-operative data.

	First generation Sunfit^®^ (*n* = 22) Novae	Second generation: Novae Sunfit TH^®^ (*n* = 128)	*p*-value	Overall cohort (*n* = 150)
**Age at surgery (years old)**				
Mean ± SD (Min–Max)	75.6 ± 11.5 (30–87)	76.5 ± 10.1 (28–90)	ns	76.0 ± 10.1 (28–90)
**Body Mass index (kg/m** ^**2**^ **)**				
Mean ± SD (Min–Max)	27.1 ± 5.4 (20–36)	27.5 ± 5.6 (18–43)	ns	27.3 ± 4.8 (18–43)
**Gender**			ns	
Female	18 (82%)	89 (70%)		107 (71%)
Male	4 (18%)	39 (30%)		43 (29%)
**Hip side**			ns	
Right	17 (77%)	73 (57%)		90 (60%)
Left	5 (23%)	55 (43%)		60 (40%)
**Diagnosis**			ns	
Primary osteoarthritis	21 (95%)	88 (69%)		109 (73%)
Aseptic osteonecrosis	1 (5%)	14 (11%)		15 (10%)
Neck fracture	0	11 (9%)		11 (7%)
Rapidly destructive osteoarthritis	0	7 (5%)		7 (5%)
Post-traumatic osteoarthritis	0	5 (4%)		5 (3%)
Dysplasia	0	3 (2%)		3 (2%)
**Approach**			ns	
Posterolateral	22 (100%)	128 (100%)		150 (100%)
**Femoral stem**			ns	
Uncemented Corail^®^ DePuy Synthes	22 (100%)	125 (98%)		147 (98%)
Cemented Fjord^®^ DePuy Synthes	0 (0%)	3 (2%)		3 (2%)
**Cup size**			ns	
43	0 (0%)	2 (2%)		2 (1%)
45	2 (9%)	5 (4%)		7 (5%)
47	3 (14%)	17 (13%)		20 (13%)
49	6 (27%)	25 (20%)		31 (21%)
51	4 (18%)	30 (23%)		34 (23%)
53	5 (23%)	27 (21%)		32 (21%)
55	1 (4.5%)	13 (10%)		14 (9%)
57	1 (4.5%)	6 (5%)		7 (5%)
59	0 (0%)	2 (2%)		2 (1%)
61	0 (0%)	1 (1%)		1 (1%)
**Prosthetic head diameter**			ns	
22.2 mm	4 (18%)	13 (10%)		17 (11%)
28 mm	18 (82%)	115 (90%)		133 (89%)

### Implants and surgery

The first group of 22 patients had a first generation of Sunfit^®^ (Stryker-Serf, Décines, France) uncemented DMC coated with alumina and HAP. The second group of 128 patients received the second-generation of Sunfit TH^®^ (Stryker-Serf, Décines, France) uncemented DMC (Figure 2). It’s made of stainless steel with a dual coating of titanium spray (thickness 150 ± 30 μm) and HA coating (thickness 70 ± 20 μm). The Acetabular Sunfit TH^®^ cup is hemispherical. The cup is anchored into the acetabular bone by mechanical fixation based on 3 anatomical points: the ischium, the ilium, and the pubis. To ensure fixation, at these 3 points, the cup presents three slightly higher ridges that divide the acetabular cup into three 120° segments. The equatorial press fit is ensured by a regular peripheral band of slightly raised ridges distributed around these 3 points (Figure 2). The polyethylene liner is five-eight of a sphere. The prosthetic head was made of Inox for all cases with a diameter of 28 mm for 133 patients (89%) and 22.2 mm for 17 others (11%).

An uncemented Corail^®^ stem (DePuy Synthes, Warsaw, IN, USA) was used for the femoral implant.

All patients were operated on by the same surgeon in one orthopedic department between September 2006 and June 2012. A standardized posterolateral surgical approach was used in all cases.

### Data assessment

Patients were evaluated preoperatively by the surgeon. All the preoperative demographic and clinical data were available in the patient file. In 2022, patients were assessed postoperatively during a phone consultation by an independent observer who recorded complications, reinterventions, modified Harris hip score (mHHS), and level of satisfaction. The last x-ray was retrieved where possible. If the patient was deceased, the patient’s trusted support person was contacted to confirm the date of death and that the patient had died with the original cup in place.

### Statistical analysis

All statistical analysis was performed using XLSTAT (2021, Addinsoft, Paris, France). Continuous variables were described using means, standard deviation, and ranges. Categorical variables were described using counts (percent). Categorical outcomes were compared using Fisher’s exact test and the chi-squared test. Normally distributed continuous variables were compared using the student *t*-test. For non-normally distributed continuous variables, the Mann-Whitney test was used. The significance threshold was set at 5%. The Kaplan–Meier method was used to estimate survival and 95% confidence intervals (CI) at 10 and 15 years for cup revision for any reason in the two groups and for the global cohort.

## Results

**Figure 3:**
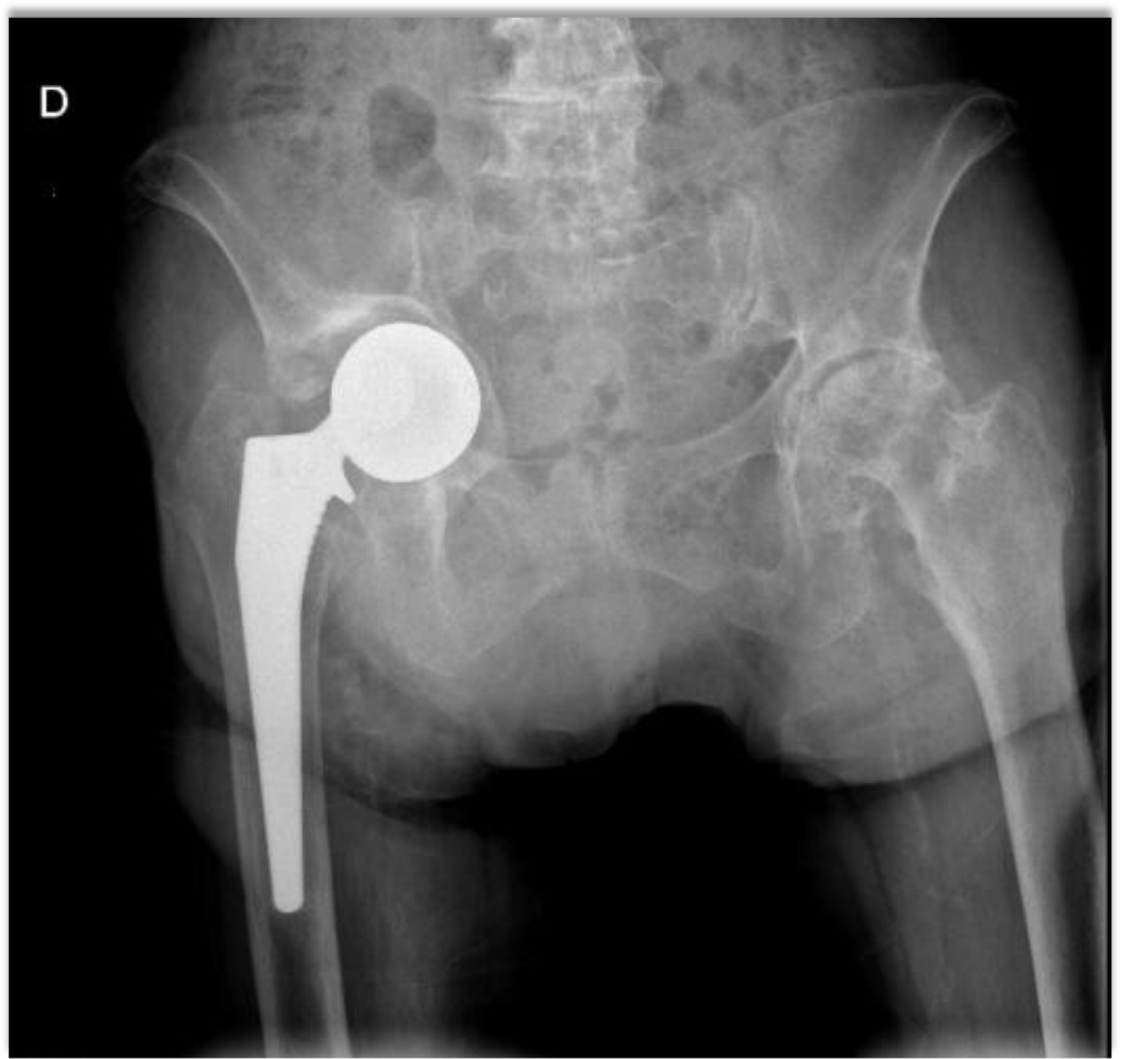
X-ray of a first generation Sunfit^®^ loosening case with secondary displacement.

At the end of the follow-up, the revision rate was 9.1% (*n* = 2/22) for the 1st generation and 1.6% (*n* = 2/128) for the 2nd generation (*p* = 0.10). In the 1st generation group, both revisions (*n* = 2/22) were performed for early aseptic loosening of the acetabulum at one and six years postoperatively (Figure 3). In the 2nd generation group, there was only one case of loosening after 8 years of follow-up (*n* = 1/128). The loosening rate is higher for the 1st generation of coating: 9.1% versus 0.78% (p = 0.056).

In the 2nd generation group, there was one case (*n* = 1/128) of extra prosthetic dislocation due to lower limb shortening and requiring femoral revision only. There were no intra-prosthetic dislocations. One postoperative infection was reported (*n* = 1/128), which was treated by reoperation with lavage, implant retention, and targeted antibiotic therapy. At the end of the follow-up, the mean modified Harris hip score was 83.2 ± 9.1 (range 57.7–100).

The level of satisfaction was very satisfactory for 86% of patients in the 1st generation group and 94% in the 2nd generation, without significant difference between both groups (Table 2).

**Table 2:** Results.

	First generation Novae Sunfit^®^ (*n* = 22)	Second generation: Novae Sunfit TH^®^ (*n* = 128)	*p*-value	Overall cohort (*n* = 150)
**Revision (acetabular or femoral)**	2 (9%)	2 (1.6%)	ns	4 (2.7%)
**Acetabular revision**	2 (9%)	1 (0.78%)	ns	3 (2%)
**Complications**			<0.0001	
Acetabular loosening	2 (9%)	1 (0.8%)		3 (2%)
Infection	0	1 (0.8%)		1 (0.7%)
Extraprothetic dislocation	0	1 (0.8%)		1 (0.7%)
Intraprothetic dislocation	0	0	0	
**Acetabular survival**				
10 years survival	90.9%	99.2%	0.009	98.0%
15 years survival	90.9%	99.2%	0.009	98.0%
**Harris Hip score (at the last FU)**		ns		
Mean ± SD (Min–Max)	84.9 ± 7.5 (67–95)	83.0 ± 9.6 (57.7–100)		83.2 ± 9.1 (57.7–100)
**Level of satisfaction**			ns	
Very satisfied	13 (59%)	90 (70%)		103 (69%)
Satisfied	6 (27%)	31 (24%)		37 (25%)
Disappointed	1 (4.5%)	5 (4%)		6 (4%)
Dissatisfied	2 (9%)	2 (2%)		4 (3%)

**Figure 4:**
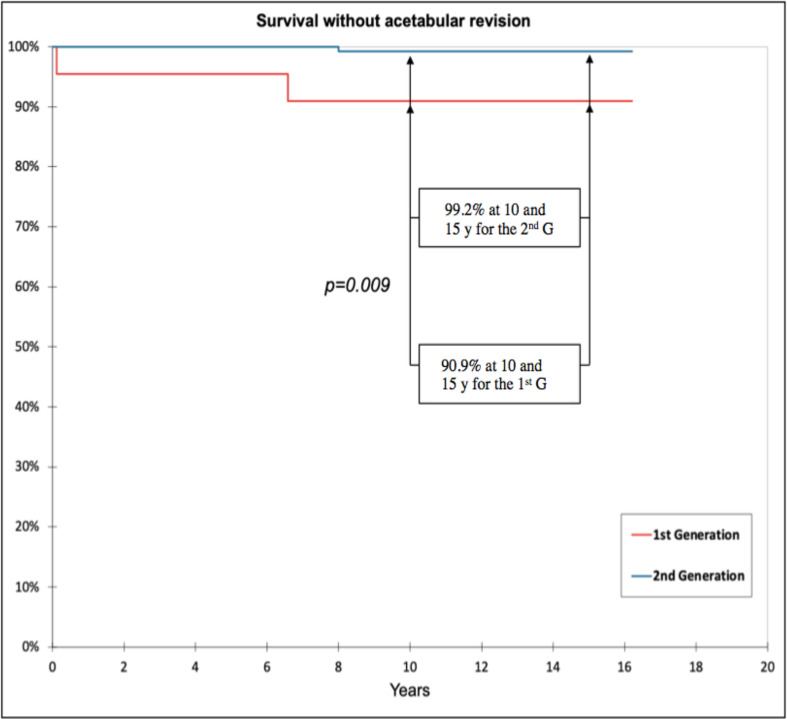
Survival without acetabular revision at 10 and 15 years for the 1st generation (90.9%) and for the 2nd generation (99.2%).

Overall survival at 10 and 15 years was 90.9% for the 1st generation and 98.4% for the 2nd generation (*p* = 0.042). Survival without acetabular revision at 10 and 15 years was 90.9% for the 1st generation and 99.2% for the 2nd generation (*p* = 0.009) (Figure 4).

## Discussion

The main finding of the present study is the excellent survival without acetabular revision at 10 and 15 years for the new generation Novae^®^ Sunfit TH Serf cups coated with HAP and plasma spray titanium (99.2%). Moreover, this survival is significantly better than the first-generation cups (*p* = 0.009).

The second-generation implants used in the present study were introduced in 2007. While the global design of the implant between Sunfit^®^ and Sunfit TH^®^ remained the same, the evolution consisted of the macrostructures and a new coating (Figure 2).

The alumina coating of the first-generation cups impacted probably the acetabular survival [4, 8, 9], with an increased risk of aseptic loosening by delamination [4]. Indeed, it has been reported that plasma-sprayed alumina did not promote osteointegration and had poor long-term properties [8, 10]. The hydroxyapatite coating is known for its osteoconductive properties, allowing excellent osteointegration and reducing osteolysis in the presence of wear particles. Nevertheless, the hydroxyapatite coating will dissolve over time as osteointegration proceeds [11–13]. That’s why titanium’s high biocompatibility and low modulus of elasticity make it an interesting agent as a surface coating [10, 14]. Titanium plasma spray, used in the second generation of implants Novae^®^ Sunfit TH (Serf) is also very rough, allowing good bone infiltration on the implant surface once the HAP has dissolved.

The change in the macrostructures of the cup’s external geometry was also beneficial. The Novae^®^ Sunfit TH has three new ridges (a few tenths of a millimeter higher) divided into three 120° segments for fixation on the ischium, ilium, and pubis, which increase equatorial press fit and, therefore, primary fixation. The presence of new grooves distributed harmoniously from the equator to the pole of Sunfit TH also improved primary fixation as well as secondary fixation by allowing the bone to integrate.

Previous studies conducted on first-generation uncemented DMC implants have shown 15-year survival rates ranging from 85.2% to 96% and aseptic loosening rates between 1% and 5.5%, probably partially explained by the alumina coating as discussed previously.

The present study confirms a significantly higher long-term survival rate with the new generation DMC compared to the first generation, attributed to design and coating changes (99.2% vs. 90.9%, respectively). These results, similar to those of other studies in the literature (Table 3), suggest that the use of second-generation uncemented DMC implants represents a reliable and safe option for active patients [18, 19].

**Table 3:** Comparison of dual-mobility cups loosening, dislocation rates and survivorship in the literature.

Authors	*n*	Mean follow-up	Cup coating	Cup aseptic loosening	Dislocation rates	Cup survivorship
Philippot et al. [6]	438	17	Alumina-HAP	3%	5%	96%
Combes et al. [15]	2179	7	HAP, alumina-HAP, titanium-HAP	1.7%	0.6%	93%
Laurendon et al. [16]	100	10	Titanium HAP	0%	0%	100%
Chouteau et al. [17]	168	8.4	Titanium HAP	0%	0%	/
Gaillard et al. [22]	163	11	Titanium HAP	0.32%	0%	98%
Present study	128	14.2	Titanium-HAP	0.78%	0.78%	99.2%

Dislocation rates reported in the literature for DMC implants vary between 0% and 2% across different series, compared to 0.3–10% for conventional implants [6, 20]. In the present study, the dislocation rate was 0.8% (*n* = 1/150), which corroborated the results found in the literature with this type of DMC [17, 21, 22]. The DMC did not have an augmentation of the dislocation rate as a function of time, as this was the case with other concepts of THA with polyethylene bearing [8, 23].

The only case of dislocation in this study was due to shortening of the lower limb requiring femoral stem revision, without acetabular revision.

There was no case of intra-prosthetic dislocation, as found in the metanalysis of Batailler et al. [20]. The intra-prosthetic dislocation became very rare with the new DMC, thanks to decreased wear and debris from cross-linked polyethylene, liner design, neck shape, and an efficient retentive system.

This retrospective study has some limitations. First, radiographs were not always available for every patient at the last follow-up. Second, the last consultation at the last follow-up was carried out by an independent surgeon by phone (complications, reinterventions, mHHS (mHHS), and level of satisfaction) if the patient was not able to come back to the hospital. However, the main objective was the survival rate and the knowledge of a potential revision, data easily collected remotely. Thirdly, the number of patients in the Novea Sunfit^®^ first generation group was low because this implant was not widely used in the center and changed quickly for the second generation.

Nevertheless, it is the first study comparing specifically two generations of a DMC from the same manufacturer, reporting long-term survival for a large cohort of uncemented DMC arthroplasties and showing excellent outcomes in the long term.

## Conclusion

The second generation of uncemented DMC granted satisfactory survival and clinical outcomes in the long term, with a high 15-year survivorship of 99.2%. Survival without acetabular revision was significantly higher at 10 and 15 years of follow-up with the second generation of DMC with plasma-sprayed titanium and HAP coating compared to the first generation of DMC coat. The dislocation was uncommon, thanks to the dual mobility concept. This second generation of uncemented DMC can be safely used in primary THA for old patients with high dislocation risk factors, as well as younger patients at risk of dislocation.

## Data Availability

The data that support the findings of this study are available on request from the corresponding author. The data are not publicly available due to their containing information that could compromise the privacy of research participants.
